# Burden and Determinants of Adverse Effects from Antiseizure Medications: Insights from Saudi Cohort

**DOI:** 10.3390/medicina62020419

**Published:** 2026-02-23

**Authors:** Bshra A. Alsfouk, Reem M. Asiri, Abdulmohsen Y. Assiri

**Affiliations:** 1 Department of Pharmaceutical Sciences, College of Pharmacy, Princess Nourah Bint Abdulrahman University, Riyadh 11671, Saudi Arabia; 2 Department of Clinical Pharmacy, Riyadh First Health Cluster, Riyadh 11622, Saudi Arabia; remasiri@moh.gov.sa; 3 Pharmaceutical Care Administration, Armed Forces Hospital Southern Region, Khamis Mushait 62413, Saudi Arabia; abdulmohsen9@hotmail.co.uk

**Keywords:** antiepileptic drugs, epilepsy, Liverpool Adverse Events Profile (LAEP), side effects of antiseizure medications (ASMs), treatment tolerability

## Abstract

*Background and objectives*: Antiseizure medications are essential for epilepsy management but often cause adverse effects that impact treatment adherence and quality of life. This study investigates the incidence rate and determinants of high-burden adverse effects of antiseizure medications. *Materials and Methods*: This study was a cross-sectional study including data extraction by a medical record review and administration of a standardized scale. It was conducted at an epilepsy outpatient clinic in Saudi Arabia and included adult patients on antiseizure medications. The validated Arabic version of the Liverpool Adverse Events Profile (LAEP) was used. The total LAEP scores ranged from 19 to 76. In this study, LAEP scores ≥ 45 were classified as high-burden adverse effects. *Results*: Of 153 included patients, 84 (54.9%) had high-burden adverse effects. The overall mean (SD) LAEP score was 45.63 (21.04). The most frequently rated adverse effects were difficulty in concentrating, with a mean score of 2.71 out of 4, followed closely by disturbed sleep (2.69), sleepiness (2.63), and memory problems (2.56). Of examined variables, generalized seizure and polytherapy were significantly associated with increased adverse effects. Likewise, uncontrolled seizure and presence of depression comorbidity were also associated with increased risk of adverse effects, but not statistically significant. *Conclusion*: The study reported a high rate of adverse effects of antiseizure medications and identified patients at high risk of adverse effects. Early recognition of these patients is important to provide appropriate care, including counselling, regular monitoring, and management of psychiatric comorbidities. Central nervous system symptoms were the most frequently reported adverse effects. Initiation of antiseizure medications with low doses and gradual titration may improve tolerability. Future research should focus on prediction adverse effects using pharmacogenomic AI-based decision-making tools.

## 1. Introduction

Epilepsy is a chronic neurological condition that affects approximately 50 million people worldwide [1]. The prevalence of epilepsy in Saudi Arabia is about 3.96 per 1000 people [2]. Antiseizure medications are the backbone of epilepsy management. There are about 30 antiseizure medications available in the US for treating epilepsy [3]. The selection of antiseizure medications depends on several factors including seizure type, comorbid conditions, adverse effects profile of antiseizure medications, and patients’ preference [4].

Adverse drug effects are defined as harmful or unpleasant effects resulting from the use of medications [5]. Adverse effects of antiseizure medications can be classified into five classes: type A (related to medication’s mechanism of action), type B (idiosyncratic reactions), type C (chronic effects), type D (delayed effects), and type E (effects due to drug interactions) [6]. type A adverse effects of antiseizure medications are mainly central nervous system symptoms such as sedative effects, coordination disturbances, memory problems, and psychiatric symptoms. These adverse effects are common, predictable, and dose-dependent [5,6,7]. On the other hand, type B effects are uncommon, unpredictable, and can be life-threatening. This type of adverse drug reaction includes skin rashes and severe cutaneous reactions, hepatotoxicity, and hematological reactions [5,6,7]. Type C adverse effects result from prolonged use of medications and cumulative dose and include osteoporosis, changes in body weight, hair loss, and gingival hyperplasia [6]. Type D effects include teratogenic and carcinogenic effects of antiseizure medications [6]. Type E adverse effects are those resulting from drug interactions and include increased toxicity or loss of effectiveness of antiseizure medications or the concomitant drugs [6].

Generally, newer antiseizure medications have improved safety profiles [8]. However, each antiseizure medication has a distinct adverse effects profile. For instance, lamotrigine and oxcarbazepine were found to exhibit more type B adverse reactions while topiramate, lacosamide, and zonisamide were associated with more type A adverse effects [9]. Understanding the tolerability profiles of antiseizure medications helps in selecting the most appropriate medication for patients [8].

Adverse effects are a leading cause of treatment failure, contributing to the early discontinuation of antiseizure medications in about 20% of patients [10], and are recognized as one of the strongest predictors of impaired health-related quality of life [11]. Furthermore, adverse drug effects often limit the ability to administer therapeutic doses [6], reducing medication adherence [12], and impose a considerable financial burden on healthcare systems [13]. Therefore, adverse effects of antiseizure medications should be measured regularly at clinical visits. Unfortunately, the literature shows that adverse effects are under-reported and under-documented. A study in Saudi Arabia evaluating the documentation of antiseizure medications’ adverse effects by clinicians found that most adverse effects were not recorded in the patients’ medical records [14]. A regional study in the United Arab Emirates recommended regular monitoring of adverse effects of antiseizure medications [15].

There are few studies in Saudi Arabia investigating the adverse drug reactions in epilepsy. The existing research has evaluated adverse effects among geriatrics [16,17], pediatrics [18], and pregnant women [19,20]. There is limited research on other populations. It is well known that the majority of adverse drug reactions are preventable [15,21]. Therefore, it is important to identify patients at high risk for adverse effects early. This study aimed to determine the incidence rate and predicators of high-burden adverse effects of antiseizure medications in adult patients in Saudi Arabia.

## 2. Methods

### 2.1. Study Design and Setting

This study was a cross-sectional study including data extraction from patients’ medical records and a questionnaire. It was conducted at the epilepsy outpatient clinic at the Armed Forces Hospital, Southern Region, Saudi Arabia. Data was collected between 10 May and 14 August 2023.

### 2.2. Participants

The study included patients on antiseizure medications for at least four weeks at the data collection date. Inclusion criteria were that patients were aged 18 years or older and had been diagnosed with epilepsy. Patients were excluded if they were taking any antiseizure medications for non-epileptic indications because the study population was limited to individuals with epilepsy, and antiseizure medications prescribed for non-epileptic conditions often differ in dosing and regimens. Also excluded were patients who could not fill out the questionnaire, including those with intellectual disability and learning difficulties. However, patients who had status epilepticus were not excluded because they would have resumed a good level of consciousness after the critical state in the intensive care unit [22].

The sample size was calculated by using an online statistical calculator (Statulator: sample size calculator for estimating a single mean) [23]. Assuming an expected population standard deviation of 8.9, obtained from a previous study conducted in Saudi Arabia on adverse drug effects of antiseizure medications [17], and using a 95% confidence level with a precision (margin of error) of 1.5, the required sample size was calculated as 139. To ensure consistency, the results were verified using the standard single-mean sample size formula [24]; $n=\frac{{\left(Z_{\alpha }\times \sigma \right)}^{2}}{e^{2}}$, where *n* is the required sample size, *Z_α_* is the *Z* score for the selected type I error, *σ* is the population standard deviation, and *e* is the margin of error. Using the same parameters in the formula yielded a similar required sample size (*n* = 135), consistent with the results from the online calculator. The convenience sampling method was used for the recruitment of the study participants.

### 2.3. Data Collection and Assessment Scale

Data collection consisted of a medical record review and administration of a standardized scale. Data was extracted from the record using a predesigned data collection form. The collected data included four parts: demographic information including gender and age; epilepsy and seizure characteristics including seizure type, date of first seizure, duration of epilepsy, seizure control status, date of last seizure, and duration of seizure freedom; antiseizure medication details including regimen (monotherapy vs. polytherapy), number of antiseizure medications used, and for each drug, name, dose, and dosing frequency; and clinical information included comorbidities and co-medications.

The Liverpool Adverse Events Profile (LAEP) [25] was used to measure the adverse effect of antiseizure medication(s). LAEP is a 19-item self-reported survey including unsteadiness, tiredness, restlessness, feeling of anger or aggression toward others, nervousness and agitation, headache, hair loss, problem with skin (e.g., acne, rash), double–blurred vision, upset stomach, difficulty in concentrating, trouble with mouth or gums, shaky hands, weight gain, dizziness, sleepiness, depression, memory problem, and disturbed sleep. It is used as a comprehensive measure of adverse effects from the antiseizure medication(s) in the past four weeks. It is used to quantify the symptoms by the patients as it is scored on a 4-point Likert scale with 4 (always a problem), 3 (occasionally a problem), 2 (rarely a problem), and 1 (never a problem). The total scores range from 19 to 76, and high scores signify a higher frequency of symptom reporting. The validated Arabic version of the Liverpool Adverse Events Profile was used, and permission was obtained from the author [17].

Patients who met the eligibility criteria and gave informed consent were asked to complete an electronic version of the study scale, reflecting their feelings over the past four weeks. Their overall LAEP score was then computed. At the same time, demographic and clinical data were collected from the patients’ electronic medical records.

### 2.4. Study Outcomes and Variables

The primary outcome in this study was to determine the rate of adverse drug effects of antiseizure medications by using the LAEP. In this study, a cutoff point for categorizing adverse effects was a LAEP score of 45. Scores below 45 were considered low-to-moderate-burden adverse effects, while scores equal to or above 45 indicated high-burden adverse effects. A total score of 45 or higher is consistent with a response pattern in which patients report many adverse effects as “sometimes” (3) or “always” (4), indicating that adverse effects are not sporadic or trivial but occur with sufficient frequency to impact daily functioning. A cutoff score of ≥45 on LAEP represented a clinically meaningful threshold that indicates a high overall burden of antiseizure-related adverse effects and aligns with prior research use. The Arabic version of the LAEP uses the same scoring system; therefore, a cutoff score of 45 remains a valid threshold [17,26,27,28,29]. The secondary outcome was to identify the determinants of high-burden adverse effects of antiseizure medications. Investigated factors were age, gender, seizure type, duration of epilepsy, seizure control status, depression, and polytherapy.

Epilepsy type was classified as generalized, focal, or unclassified based on the most recent seizure and epilepsy classification guidelines from the International League Against Epilepsy (ILAE) [30]. The duration of epilepsy was measured from the date of first seizure to the date of data collection. Seizure status was considered controlled if the patient had remained seizure-free for a minimum of 12 months at the time of data collection; otherwise, it was classified as uncontrolled. Depression was identified based on documented clinical diagnosis and the recorded use of antidepressant medications in patient medical records.

### 2.5. Data Analysis

Data was analyzed using Statistical Package for Social Sciences (SPSS) version 21 (SPSS Inc., Chicago, IL, USA) and GraphPad Prism 10.4.2 (GraphPad Software, San Diego, CA, USA). Descriptive analysis was presented as frequency and percentage while numerical variables were presented as mean and (standard deviation, SD). The associations between high-burden adverse reactions of antiseizure medications (dependent variable) and several potential predictors (independent variables) were assessed by multiple logistic regression analysis. The dependent variable in the regression analysis was a binary variable (i.e., low-moderate vs. high-burden adverse drug effects): patients with LAEP scores of ≥ 45 were classified as “high-burden” while those with scores of < 45 were categorized as “low-moderate”. Regression analysis was conducted using adjusted odds ratios (ORs) with 95% confidence intervals (CIs) for the variables under investigation. Age was a continuous variable, whereas all other factors were analyzed as binary categorical variables. Monotherapy regimens were analyzed as a single reference category and not stratified by antiseizure medication class. Dual and triple therapies were combined into a single polytherapy category in the regression model. Statistical significance was defined as a *p*-value of less than 0.05.

### 2.6. Ethical Consideration

The Institutional Review Board at Armed Forces Hospital, Southern Region, Saudi Arabia, approved this study with IRB Registration Number AFHSRM- REC/2023/PHARMACYY/680, date 8 May 2023. Good clinical practice guidelines and the Helsinki Declaration were adhered to. For ethical reasons, informed consent had been received from the patients before participating in the study. The participants were fully informed that participation was voluntary, and they had the right to withdraw from the study at any time with no consequences. Throughout the whole study, patient confidentiality was maintained. The dataset included no personal identifiers, and access was restricted to the research team using physical and IT security.

## 3. Results

### 3.1. Demographic and Clinical Characteristics of Patients

A total of 153 patients were included in this study. As demonstrated in Table 1, patients’ ages ranged from 18 to 65 years, with a mean age (SD) of 35.2 (12.5) years. Males were slightly predominant (58.2%). Approximately 53% of the patients had generalized seizures with a duration of epilepsy of ~8 years on average. About 51% of the patients were seizure-free and their epilepsy was controlled.

### 3.2. Antiseizure Medication Profiles in the Study Cohort

As described in Table 2, antiseizure medication monotherapy was taken by 57.5% of the study patients. The most commonly prescribed monotherapy drugs were levetiracetam, valproate, carbamazepine, and lamotrigine.

A total of 65 patients received polytherapy, with 58 on dual regimens and 7 on triple regimens (Table 3).

**Figure 1:**
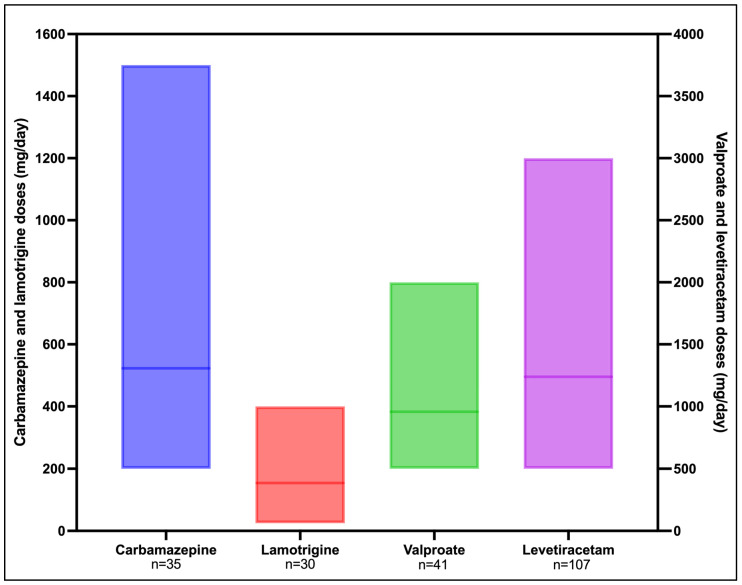
Dosages of antiseizure medications. Bar represents minimum to maximum range; middle line indicates the mean.

The doses of the most commonly prescribed medication antiseizure medications are summarized in Figure 1.

### 3.3. Rate of Adverse Effects of Antiseizure Medications

**Figure 2:**
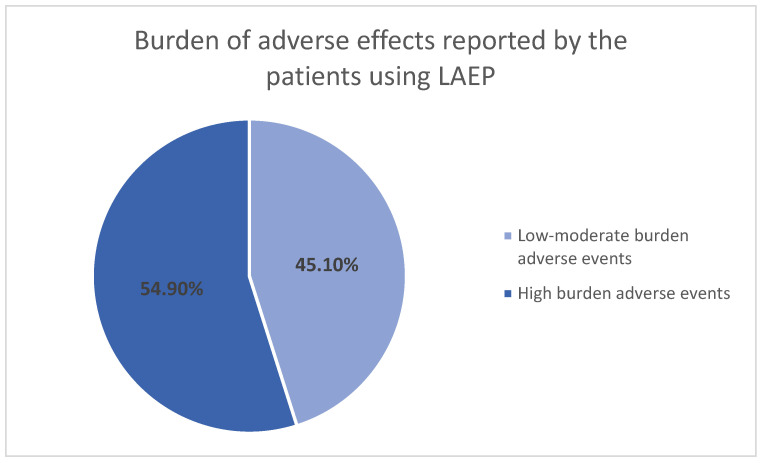
Rate of adverse effects of antiseizure medications reported by the study patients using the Liverpool Adverse Events Profile (LAEP). Low-moderate-burden represents LAEP scores < 45, high-burden indicates scores ≥ 45.

The rate of adverse effects of antiseizure medications was measured using the self-administered LAEP scale. A total LAEP score was calculated for each patient. Based on these scores, adverse effects were classified into two categories: low-to-moderate burden (LAEP score < 45) and high-burden (LAEP score ≥ 45). As shown in Figure 2, 69 patients (45.1%) reported low-moderate-burden adverse events, while 84 patients (54.9%) experienced high-burden adverse events from antiseizure medications. The overall mean (SD) LAEP score was 45.63 (12.81).

The average scores for each adverse effect of the 19 LAEP items are shown in Table 4. The most frequently rated adverse effects were difficulty in concentrating, followed closely by disturbed sleep, sleepiness, memory problems, hair loss, nervousness and agitation, depression, and upset stomach. The least reported adverse effects were problems with the skin including acne and rash, tiredness, weight gain, and trouble with mouth and gums.

### 3.4. Determinants of Adverse Effects of Antiseizure Medications

Multivariable logistic regression analysis was conducted to investigate the relationship between adverse effects and several factors. Examined variables were age, gender, seizure type, duration of epilepsy, seizure control status, depression comorbidity, and antiseizure medication regimen. The results are shown in Table 5. Factors significantly associated with increased adverse effects were generalized seizures and polytherapy. Of patients on polytherapy regimens, 63.1% experienced high-burden adverse effects from antiseizure medications in comparison to 48.9% of those on monotherapy regimens (*p* = 0.048).

Uncontrolled seizure and presence of depression comorbidity were also associated with increased risk of adverse effects, but not statistically significant. Fifty-one (68%) patients with uncontrolled seizures experienced high-burden adverse effects from antiseizure medications compared to 33 (42%) of those with controlled seizures. The risk of having adverse effects in patients with uncontrolled seizure was twice (OR = 2) that in seizure-free patients. Likewise, patients with depression tended to report the adverse effects more than patients with no depression, 66.6% vs. 53.9%, respectively.

Mean LAEP scores (SD) across clinical and demographic variables are summarized in Supplementary Table S1.

## 4. Discussion

This study aimed to determine the rate of adverse drug effects associated with antiseizure medications among adult patients with epilepsy in Saudi Arabia. In this study, the rate of high-burden adverse effects (LAEP ≥45) was 54.9% with an overall mean LAEP score of 45.63. This was consistent with the incidence rate reported by other studies [31,32]. However, the previous study in Saudi Arabia by Alruthia et al. [17] observed a lower rate with a mean LAEP score of 28.9. This could be because they included patients on monotherapy only. Furthermore, other studies reported lower incidence rates of adverse effects of antiseizure medications compared to our study [28,29]. Several factors could contribute to this variation in the rate of adverse effects of antiseizure medications among studies including variation in prescribed antiseizure medications (type, dose, titration), regimen used (monotherapy vs. polytherapy), settings (outpatients vs. inpatients), rate of refractory seizures, and differences in patients’ characteristics. In fact, measuring adverse effects by a scale was found to be associated with higher rates of adverse effects compared to those detected by spontaneous reporting or unstructured interview. A multicenter study that included 809 patients with refractory seizure demonstrated that the rate of adverse effects detected by a scale was three times larger than that measured by an unstructured interview, 93% vs. 36%, respectively [33].

Factors associated with increased rates of adverse drug effects of antiseizure medications in the present study were generalized seizure, polytherapy antiseizure medication regimen, uncontrolled seizure, and presence of depression comorbidity. Our study showed that patients with generalized seizures had a significant association with adverse drug effects. However, the association was not found to be consistent other studies [29,34]. The findings of this study demonstrated that polytherapy had a significant association with adverse effects. This is consistent with the findings of several studies that showed that polytherapy was a variable that influenced adverse effects [10,35,36]. Clearly, administration of two or more antiseizure medications increases the risk of developing adverse effects due to the probability of pharmacokinetic and pharmacodynamic drug interactions. However, other studies report contradictory results. Canevini et al. [33] did not find differences in adverse effects between monotherapy and polytherapy groups. The possible justification, as stated by the authors, was due to personalization of antiseizure medication regimens by physicians. Joshi et al. [37] showed there was no significant difference in adverse effect between monotherapy and combination of two antiseizure medications, whereas a combination of three antiseizure medications or more was associated with higher adverse effects. In the present study, patients with uncontrolled seizures (i.e., lack of 12-month seizure freedom) had increased adverse effects compared to those with controlled seizures. Although the association did not reach statistical significance, it may still be clinically relevant. This is in line with the findings reported by Kowski et al. [29], who found that lack of 12-months seizure freedom was an independent predictor for a high adverse effects burden (LAEP ≥ 45). Likewise, the increased number of seizures was found to be a predictor of adverse drug effects in epilepsy [34]. One possible explanation for this finding is that patients with uncontrolled seizures often need larger doses and multiple antiseizure medications in an attempt to manage their intractable epilepsy, which in turn is associated with increased adverse effects. In this study, patients with depression tended to report that adverse effects were more likely than patients with no depression. In fact, the existing literature consistently shows a correlation between depression and adverse drug effects [38,39,40]. Panholzer et al. [28] demonstrated that depression significantly influenced total LAEP scores as well as individual items, affecting both emotional and somatic symptoms domains. This information is important in clinical practice because changing antiseizure medications regimens differ depending on whether the adverse effects are attributed solely to antiseizure medication or confounded by depression. Furthermore, it is important to screen for depression in all people with epilepsy. Treating depression may increase the tolerability of antiseizure medications [28]. In the regression analysis, seizure control and depression were assessed as binary variables. Dichotomization of variables was chosen to enhance clinical relevance and simplicity. Categorization of seizure control as seizure-free versus not is more clinical meaningful than seizure frequency. Similarly, patient-reported depression offers a more meaningful, patient-centered measure than scaled assessments. Additionally, binary outcomes simplify modeling, improve interpretability of effect sizes (e.g., odds ratios, risk difference), and support clearer communication of results to clinicians, patients, and policymakers.

The most frequently rated adverse effects in the presented study were difficulty in concentrating, disturbed sleep, sleepiness, and memory problems. This is consistent with findings from other studies. Budikayanti et al. [35] observed that tiredness, sleepiness, memory problems, and difficulty in concentrating were the most common adverse effects of antiseizure medications in their cohort. A study in Saudi Arabia found that drowsiness was the most frequently reported adverse effect [14]. Generally, CNS adverse effects of antiseizure medications are common and related to their pharmacological effects. Antiseizure medications are CNS depressants, and their mechanisms of action involve reducing brain overexcitability to suppress seizures. This interferes with normal neurotransmission and brain functions including alertness and cognition, resulting in adverse effects including difficulty in concentrating, sleepiness, and memory problems [7]. The CNS adverse effects can be minimized by titer and adjusting the dose with a “start low, go slow” approach allowing tolerance to develop [6]. Also, it is important to educate patients about these expected adverse effects. Neuropsychiatric symptoms including nervousness or agitation, depression, and anger or aggression were among the most frequently reported adverse effects in the study. This may be attributed to the predominance of levetiracetam, both as monotherapy and in combination regimens. Additionally, the potential overlap between depressive symptoms and certain LAEP items including sleep disturbance, concentration, and fatigue may introduce confounding effects.

Using a validated and reliable tool (i.e., LAEP) is a strength of this study. LAEP [25] is a systematic measure of adverse effects specifically developed for antiseizure medications. It is a graded scale and can quantify the symptoms. However, as a self-reported scale, it imposes disadvantages including subjectivity and the possibility of over-reporting adverse effects. This study focused on epilepsy. Even if other indications were not investigated, the results can certainly guide clinical practice with regards to the tolerance to antiseizure medications and the choice in clinical practice [41,42]. The study has several limitations. First, the single-center outpatient setting may limit generalizability of the findings to other epilepsy populations and healthcare systems. Second, the cross-sectional design permits assessment of associations between antiseizure medication adverse effects and clinical factors but does not allow causal inference. Additionally, the relatively short recruitment period (10 May to 14 August 2023), which was primarily due to researcher availability for data collection, may have introduced selection bias and limit representativeness of the outpatient epilepsy population. The sample size (n = 153), relative to the number of predictors included in the multivariable model, raises the possibility of overfitting. Furthermore, the small number of patients receiving triple therapy (n = 7) limits statistical power to assess differential adverse-effect burden by polytherapy complexity. In the present study, approximately 23% of patients were taking co-medications. This may increase the likelihood of drug–drug interactions between antiseizure medications and other drug classes [43], potentially contributing to a higher risk of adverse effects.

Future research on adverse effects of antiseizure medications should focus on better predication of adverse effects by integrating pharmacogenomic and precision medicine. The association between polymorphisms in drug metabolizing enzymes, drug targets, and drug transporter proteins with specific antiseizure medication adverse reactions needs more investigation in the Saudi population to guide safer antiseizure medications selection [44]. Furthermore, more research is required to help in implementing decision-making AI tools in health systems that can assist prescribers to choose the better tolerated antiseizure medications and minimize adverse reactions [45].

## 5. Conclusions

This study found a high rate of adverse effects of antiseizure medications, with half of the patients experiencing a considerable burden of adverse effects. This study also identified high-risk patients–those with generalized seizures, on polytherapy, having uncontrolled seizures, and having depression–were more likely to report adverse effects. It is important to recognize patients at high risk of adverse effects early and provide them with appropriate care including counselling, frequent monitoring of adverse effects, and assess and manage psychiatric comorbidity. The most frequently reported adverse effects were CNS symptoms including tiredness, sleepiness, memory problems, and difficulty in concentrating. Starting antiseizure medications at a low dose with gradual up-titration may allow tolerance of many CNS adverse effects. Future research directions in this area involve investigating better prediction of adverse effects by integrating pharmacogenomic as well as decision-making AI tools.

## Figures and Tables

**Table 1:** Demographic data of study patients (n = 153).

Variable Description	Frequency (n)	%
Age (years)		
Mean ± SD	35.2 ± 12.5	
Range	18–65	
Gender		
Male	89	58.2%
Female	64	41.8%
Seizure type		
Focal	62	40.5%
Generalized	81	52.9%
Combined	10	6.5%
Duration of epilepsy		
<5 years	54	35.3%
5–10 years	56	36.6%
>10 years	43	28.1%
Mean ± SD (months)	95.2 ± 80.8	
Epilepsy status		
Controlled	78	50.9%
Uncontrolled	75	49.1%
Duration since last seizure		
<12 months	75	49.0%
12–36 months	35	22.9%
>36 months	43	28.1%
Mean ± SD (months)	26.9 ± 30.9	

Out of 153 patients, 128 (83.7%) had no comorbidities while the remaining 25 (16.3%) patients had at least one comorbidity. The most reported comorbidities were hypertension (n = 20), diabetes mellitus (n = 17), depression (n = 12), and cardiovascular diseases (n = 11). A total of 35 patients (22.9%) were taking co-medications, with 23 of them receiving co-medications that affect the central nervous system (CNS).

**Table 2:** Antiseizure medications taken by study patients (n = 153).

Antiseizure Medications	Count (n)	%
Antiseizure medication regimen		
Monotherapy	88	57.5%
Polytherapy	65	42.5%
Number of antiseizure medications		
One drug	88	57.5%
Two drugs	58	37.9%
Three drugs	7	4.6%
Monotherapy antiseizure medication		
Levetiracetam	54	61.4%
Sodium valproate	15	17.0%
Carbamazepine	9	10.2%
Lamotrigine	6	6.8%
Topiramate	4	4.6%
Daily dose frequency of monotherapy		
OD, once per day	12	13.6%
BID, twice per day	76	86.4%

**Table 3:** Polytherapy antiseizure medication regimens.

Antiseizure Medications	Count (n)
Dual therapy	
Levetiracetam and carbamazepine	17
Levetiracetam and valproate	16
Levetiracetam and lamotrigine	12
Valproate and lamotrigine	5
Others	8
Triple therapy	
Levetiracetam, lamotrigine, and carbamazepine	3
Levetiracetam, carbamazepine, and valproate	2
Levetiracetam, valproate, and topiramate	2

**Table 4:** Mean (SD) and median (IQR) scores of the 19 LAEP items as rated by patients. The values were out of 4.

LAEP Items	Mean (SD)	Median (IQR)
Unsteadiness	2.24 (1.03)	2 (1–3)
Tiredness	2.15 (1.03)	2 (1–3)
Restlessness	2.43 (1.07)	3 (1–3)
Feeling of anger or aggression toward others	2.49 (1.14)	3 (1–3)
Nervousness or agitation	2.52 (1.08)	3 (2–3)
Headache	2.46 (1.05)	3 (2–3)
Hair loss	2.58 (1.22)	3 (1–4)
Problem with skin (e.g., acne, rash)	2.03 (1.09)	2 (1–3)
Double–blurred vision	2.21 (1.06)	2 (1–3)
Upset stomach	2.5 (1.05)	3 (2–3)
Difficulty in concentrating	2.71 (1.14)	3 (2–4)
Trouble with mouth or gums	2.16 (1.13)	2 (1–3)
Shaky hands	2.34 (1.14)	2 (1–3)
Weight gain	2.16 (1.16)	2 (1–3)
Dizziness	2.29 (1.12)	2 (1–3)
Sleepiness	2.63 (1.13)	3 (2–4)
Depression	2.5 (1.15)	3 (1–3)
Memory problem	2.56 (1.2)	3 (1–4)
Disturbed sleep	2.69 (1.2)	3 (2–4)
Overall score	45.63 (12.81)	46 (37–54)

Abbreviations: LAEP: Liverpool Adverse Events Profile, IQR: interquartile range.

**Table 5:** Multiple logistic regression analysis for predictors of adverse effects of antiseizure.

Variable Description (n = 153)	High-Burden Adverse Effects (n = 84) Frequency (%)	Adjusted OR (95% CI)	*p*-Value
Age (years)			
		1.01 (0.50–2.04)	0.972
Gender			
Female (n = 64)	36 (56.3%)	1.08 (0.54–2.15)	0.832
Male (n = 89)	48 (53.9%)		
Generalized seizure			
Yes (n = 81)	47 (58%)	1.42 (1.10–2.57)	**0.046**
No (n = 72)	37 (51.4%)		
Duration of epilepsy			
<5 years (n = 54)	33 (61.1%)	0.81 (0.50–1.31)	0.385
5–10 years (n = 56)	29 (51.8%)		
>10 years (n = 43)	22 (51.2%)		
Seizure control status			
Uncontrolled (n = 75)	51 (68%)	2.01 (0.45–8.94)	0.357
Controlled (n = 78)	33 (42.3%)		
Depression comorbidity			
No (n = 141)	76 (53.9%)	0.71 (0.19–2.7)	0.616
Yes (n = 12)	8 (66.6%)		
Antiseizure medication(s) regimen			
Polytherapy (n = 65)	41 (63.1%)	1.63 (1–3.47)	**0.048**
Monotherapy (n = 88)	43 (48.8%)		

Abbreviations: OR: odds ratio, CI: confidence interval. Bold *p*-values are significant (<0.05).

## Data Availability

The data presented in this study are available on request from the corresponding author. Reem Asiri has full access to the study data.

## Supplementary Materials

The following supporting information can be downloaded at: https://www.mdpi.com/article/10.3390/medicina62020419/s1, Table S1. Mean Liverpool Adverse Events Profile (LAEP) scores (SD) across clinical and demographic variables.
