# Supplementary material for: Burden and Determinants of Adverse Effects from Antiseizure Medications: Insights from Saudi Cohort
Source: Medicina (Kaunas). 2026 Feb 23;62(2):419. doi: 10.3390/medicina62020419 (PMC12942135; doi:10.3390/medicina62020419)
Supplement: Supplementary file 1 [file medicina-62-00419-s001.zip › medicina-4091331-supplementary.pdf]

Supplementary Table S1. Mean Liverpool Adverse Events Profile (LAEP) scores (SD) across clinical and demographic variables

Variable description	LAEP score Mean (SD)
Gender	
Female	46.98 (12.47)
Male	44.65 (13.03)
Generalized seizure	
Yes	45.89 (12.12)
No	45.33 (13.62)
Duration of epilepsy	
< 5 years	46.63 (14.35)
5-10 years	44.64 (13.42)
> 10 years	45.65 (9.79)
Seizure control status	
Uncontrolled	49.2 (12.66)
Controlled	42.28 (12.1)
Depression comorbidity	
No	45.28 (12.76)
Yes	49.75 (13.19)
Antiseizure medication(s) regimen	
Polytherapy	48.06 (12.1)
Monotherapy	43.83 (13.09)
